# Neurofeedback for tinnitus: study protocol for a randomised controlled trial assessing the specificity of an alpha/delta neurofeedback training protocol in alleviating both sound perception and psychological distress in a cohort of chronic tinnitus sufferers

**DOI:** 10.1186/s13063-020-04309-y

**Published:** 2020-05-05

**Authors:** Martin Jensen, Eva Hüttenrauch, Jennifer Schmidt, Gerhard Andersson, Mira-Lynn Chavanon, Cornelia Weise

**Affiliations:** 1 Division of Clinical Psychology and Psychotherapy, Department of Psychology, Philipps University Marburg, Gutenbergstrasse 18, 35032 Marburg, Germany; 2 HSD Hochschule Döpfer, University of Applied Sciences, Waidmarkt 3 und 9, DE-50676 Köln, Germany; 3 Department of Behavioural Sciences and Learning, Linköping University, Linköping, Sweden; 4 Department of Clinical Neuroscience, Karolinska Institute, Stockholm, Sweden; 5 Department of Psychology, Clinical Child and Adolescent Psychology, Philipps University Marburg, Marburg, Germany

**Keywords:** Neurofeedback, Chronic tinnitus, Severe tinnitus, Tinnitus patient, Electroencephalography, Tinnitus treatment

## Abstract

**Background:**

Tinnitus is a particularly common condition and can have debilitating psychological consequences for certain people. Although several interventions have been helpful in teaching individuals to better cope with tinnitus, no cure exists at present. Neurofeedback is an emerging treatment modality in tinnitus. Previous studies, utilising an alpha/delta training protocol, have shown promise. However, they were characterised by small sample sizes and a lack of neurofeedback control conditions. Therefore, the aim of this study is to investigate whether an alpha/delta neurofeedback training protocol, compared to beta/theta neurofeedback or a diary control group, is effective in reducing not only the tinnitus sound perception but also the psychological symptoms associated with the condition.

**Methods:**

The study is designed as a three-armed randomised controlled trial. Participants are randomly assigned to a) an established neurofeedback protocol for tinnitus (alpha/delta training), b) an active control group (beta/theta training) or c) a diary control group. In the 4-week intervention period, participants in both neurofeedback groups undergo 10 sessions, whereas participants in the diary control group complete a bi-weekly diary. The primary outcomes are between group differences in tinnitus sound perception change, as measured with the Tinnitus Magnitude Index (TMI), and changes in tinnitus distress, measured with the Tinnitus Handicap Inventory (THI), 4 weeks after the start of the intervention. Secondary outcome measures include changes in tinnitus distress, sleep quality, depressive symptoms and whether neurofeedback leads to specific power changes in the trained frequency bands.

**Discussion:**

This is the first randomised controlled trial examining the efficacy of an alpha/delta neurofeedback training protocol in reducing tinnitus sound perception and the distress associated with the condition. Compared to former studies, the present study is designed to assess both the specificity of an alpha/delta neurofeedback training protocol by including an active comparator and beta/theta neurofeedback training, in addition to controlling for placebo effects by the inclusion of a diary control group. This study aims to contribute to an understanding of the influences of both specific and non-specific effects in neurofeedback treatment for tinnitus.

**Trial registration:**

ClinicalTrials.gov: NCT03550430. Registered on 27 May 2018.

## Introduction

Tinnitus, the perception of sound in the absence of external acoustic stimuli [1], is a rather common condition. However, getting a precise prevalence estimate poses a challenge. This is largely due to the heterogeneity of tinnitus definitions in research [2]. Nevertheless, prevailing research points to an estimated 10 to 20% of the general population experiencing tinnitus [2, 3]. Although most manage to live well with—and tolerate—the condition, for 2 to 3% of the population, the condition is so severe that it seriously interferes with their quality of life [2, 4]. Feelings of despair, hopelessness, anxiety and depression are commonly reported by distressed tinnitus patients, as are concentration difficulties and insomnia [5, 6].

Prior to advances in neuroscience, models in tinnitus emphasised peripheral auditory structures as the locus of tinnitus generation (e.g., [7]). Although tinnitus is likely precipitated by damage to the cochlea and/or auditory structures, current theories and models hold that it is generated and perpetuated in the brain [8] as basic assumptions. In individuals with tinnitus, spontaneous resting-state brain activity has been found to differ from that of healthy controls. For the interested reader, [9, 10] provide very readable, recent and updated accounts of neurophysiological models of tinnitus. Given the focus of the present study, the findings from neuroimaging studies, such as those outlined in, for example, [9], are important and have led to the development of several experimental neuromodulation techniques, ultimately seeking to redress the imbalances in aberrant neuronal activity. One such technique, which in the past decade has spawned considerable research activity, is neurofeedback [10]; generally, the goal of neurofeedback is the promotion of healthier brain wave patterns (e.g., [11]). Normally, brain wave activity occurs outside of conscious awareness, but by presenting it in an audio- and or visual format to a neurofeedback trainee, he/she has the possibility of influencing it, ultimately achieving more optimal brain functioning (11).

In the past decade, one finding of differences in resting state brain activity between individuals with tinnitus and healthy controls has given rise to significant experimental activity in the field of neurofeedback. In individuals with tinnitus, compared to healthy controls, reduced alpha and increased delta brain wave activities over temporal regions were observed [12]. This led to the development of neurofeedback training protocols aimed at reversing this abnormal brain activity pattern in individuals with tinnitus [13]. Using an alpha/delta ratio (ADR) training protocol, with the goal of simultaneously uptraining alpha- and down-training delta activity, three studies have arrived at similar conclusions regarding the effectiveness of neurofeedback: participants in all studies report improvement in their overall psychological well-being upon completion of neurofeedback training [9, 13, 14]. Moreover, the loudness perception also seemed to be influenced by the training when rated immediately upon completion of the intervention [13, 14]. Over time, however, this effect appeared to be less robust compared to the psychological benefits of neurofeedback and returned somewhat to baseline [9]. In summary, the results of ADR neurofeedback training on the distressing aspects of tinnitus are encouraging. Nevertheless, in these studies, as in neurofeedback studies in general [15], the influence of non-specific factors are largely ignored or otherwise not accompanied with controls. In neurofeedback, this neglect is particularly critical, as factors such as expectancy, motivation, therapist interaction and demand characteristics are assumed to play a significant, if not decisive role [9, 15–17]. Some (e.g., [16]) even consider these effects to be the healing mechanisms in neurofeedback.

One way to address this concern is to demonstrate a correlation between the self-regulation of brain activity and behavioural outcomes, i.e., outcome specificity [18]. In all of the studies assessing the effectiveness of the ADR training protocol on tinnitus, outcome specificity was reported, but with mixed conclusions. Dohrmann and colleagues [13] identified a significant correlation between changes in the ADR and tinnitus intensity reduction. This association was not significant for the correlation between the ADR and tinnitus distress. Conversely, Crocetti and colleagues [14] found changes in the ADR to be significantly associated with improvement in well-being, whereas for intensity perception, no association was observed. Lastly, Güntensperger and colleagues [9] reported outcome specificity to be mainly associated with increments in alpha frequency band activity. Overall, taking them at face value, the three studies demonstrate some elements of outcome specificity, which in turn speaks in favour of the reduced influence of non-specific effects on the outcome. However, ruling that non-specific effects were indeed the agents of change in these studies is premature when taking into consideration that 1) non-specific effects can lead to changes in brain activity [19], and more importantly, that 2) alpha frequency band activity is particularly susceptible to the influence of non-specific effects [15]. Hence, when changes in brain wave frequency bands, particularly the alpha band, are observed in neurofeedback studies, this treatment effect may wrongly be attributed to a specific effect of the treatment, when in fact non-specific effects may equally account for it.

The goal of the present study is thus to advance our knowledge of the influence of specific versus non-specific effects in ADR neurofeedback training for tinnitus. To ensure comparability with previous studies (i.e., [9, 13, 14]), a number of parameters are identical (see Methods section). However, two control conditions have been added to the study to explore whether the healing mechanisms of neurofeedback for tinnitus are indeed specific to ADR neurofeedback or whether other effects can account for the improvement in tinnitus sufferers’ well-being. The first of these control conditions is a beta/theta ratio (BTR) neurofeedback intervention. The decision to include this is informed by researchers (e.g., [15]) who have suggested that the only way to assess the efficacy of a particular neurofeedback training protocol (in this case, ADR) is to subtract the effects of an identical protocol while barring the neural targets (in this case, BTR). Although no neurofeedback study for tinnitus has used the specific BTR training protocol to date, this protocol has been used in studies aimed at enhancing attention [20], both in clinical (e.g., [18, 21, 22]) and in healthy (e.g., [20]) populations. Because all factors other than the trained frequency bands are identical between ADR and BTR, we assumed this control condition to be the most suitable to effectively control for non-specific participant factors in the present study. The diary control intervention comprises the third arm of the study. In this section, components are included that are intended to promote or enhance non-specific effects, such as expectancy, motivation, demand characteristics and the therapeutic alliance. By subtracting the influence of these from those of the neurofeedback groups, an estimate of the magnitude of non-specific effects in neurofeedback can be gauged.

Our hypotheses are based on the following arguments. First, ADR neurofeedback training was developed specifically to restore an imbalance in excitatory/inhibitory neuronal activity in the auditory cortex in individuals with tinnitus [13] and, therefore, should be more efficacious than BTR neurofeedback. Second, the ADR protocol has been used with success to reduce distress and/or intensity in three prior studies [9, 13, 14]. Third, given the added influence of non-specific effects in neurofeedback, participants in the ADR and BTR groups will have greater expectations of treatment outcomes than participants in the diary control condition. Based on these observations, we hypothesise the following:

1. Participants undergoing ADR neurofeedback training will report significantly stronger reductions in tinnitus distress and intensity from pre- to post-treatment compared to participants undergoing BTR neurofeedback.
2. Participants in both the ADR and BTR neurofeedback interventions will improve more significantly in measures of tinnitus distress and intrusiveness from pre- to post-treatment Methods/Design

### Study Design

The study, a three-armed, randomised controlled trial, will be conducted at Philipps University in Marburg, Germany. The study has been approved by the respective ethics committees of the Department of Psychology and the Department of Medicine at Philipps University with registration numbers 2018-4 k (Additional file 2) and 162/18 (Additional file 3), respectively. Furthermore, the trial is registered with clinicaltrials.gov (ClinicalTrials.gov Identifier: NCT03550430).

Sample size calculations were based on a repeated-measures multivariate analysis of variance (MANOVA) and conducted with G*Power 3.1.9.2 [23].. The total sample size needed to detect a medium assessment-by-treatment interaction effect (*f* = 0.25, *Beta* = 0.80, *Alpha* = .05, Groups = 3, measurements = 3) is 98. However, to compensate for an estimated dropout rate of 20% during treatment, the aim is to recruit 120 participants, i.e., 40 participants per group. Post-hoc analyses are carried out between ADR vs. BTR, ADR vs. diary control and BTR vs. diary control.

### Inclusion and exclusion criteria

Participants are eligible for inclusion if they meet the following criteria:

1. Age + 18 years
2. Chronic, subjective tinnitus with a duration of ≥ 6 months
3. At least mild tinnitus distress, corresponding to a score of ≥ 18 on THI [24]
4. Signed informed consent

The study’s exclusion criteria are as follows:

1. Objective tinnitus, either vascular or non-vascular in origin
2. Acute inflammatory disease of the ear/ears
3. Conductive hearing loss
4. Blockage of the ear canal by, for example, cerumen, where removal is not desired by the individual
5. Any current treatment for tinnitus
6. Severe mental health issues (moderate/severe depression, bipolar disorder, psychosis)
7. ADHD
8. Current use of a psychotropic drug for a mental health condition
9. Substance abuse
10. Previous or current neurofeedback treatment
11. Previous or current neurological conditions (e.g., a history of seizures, brain tumour, haemorrhage/stroke).

### Procedure/trial protocol

Recruitment for the trial is completed primarily through advertisements placed in local and regional media. In addition, a referral programme is established to entice ear-nose-throat physicians (ENTs) to forward participants to the study. Interested individuals can read more about the study on a dedicated website, where they also can register their interest in trial participation. Both the website and the questionnaires are provided via the Iterapi platform [25].

**Fig. 1:**
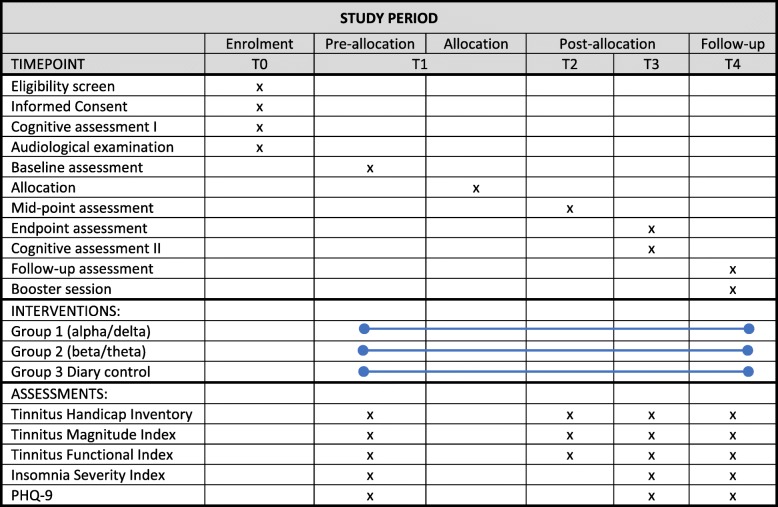
Schedule of enrolment, interventions and assessments

The enrolment period (t0, see Fig. 1) is a four-stage process. In the first stage, participants complete a battery of online screening questionnaires. These are principally designed to sort individuals into eligible and non-eligible categories, based on the inclusion/exclusion criteria. Prior to taking the screening questionnaires, participants will give informed consent for questionnaire completion only. For principally eligible individuals, the second stage in the screening process is a telephone interview. The purpose of this is to validate questionnaire responses and to assess their motivation to participate in such a trial. The third stage in t0 is a visit to the Department of Psychology. The first part of this visit provide participants with detailed information about the study, its procedures and how to complete baseline assessments, including primary and secondary outcome measures on the secure online platform. Following this, participants sign the informed consent form (see Additional file 4) and complete the State Anxiety Inventory online (STAI) [26]. Participants then complete two attention tests, the Attention Network Task (ANT) [27] and the Sustained Attention to Response Task (SART) [28], referred to in Fig. 1 as the cognitive assessment.

The fourth and final stage in t0 is a standard audiological examination at the ENT-clinic at the University Hospital of Giessen and Marburg (UKGM). There are two main purposes for this visit: The first is to rule out possible non-subjective causes of tinnitus, and the second, to acquire audiometric data. At the clinic, participants first learn about the components of the audiological examination before signing the informed consent form (see Additional file 5). Subsequently, the audiological examination, in which the below information is acquired, takes place:

1. Standard Audiogram (to 20.000 Hz)
2. Tympanometry
3. Otoacoustic emission (OAE)
4. Tinnitus loudness discomfort level
5. Tinnitus matching (loudness, pitch)

Participants who are included after the visit to the ENT clinic take the baseline assessment in the pre-allocation (t1) stage. Upon completion of this assessment, participants move to the allocation (t1) stage and are randomly assigned to one of three arms in the study: Group 1, which receives alpha/delta neurofeedback training; Group 2, which receives beta/theta neurofeedback; and Group 3, which is the diary control group.

The post-allocation (t2) intervention period lasts 4 weeks. Within this timeframe, ten neurofeedback training sessions are undertaken for the two neurofeedback groups, with a minimum of two and maximum of three sessions per week. For the diary control group, two face-to face visits to the department and two telephone calls are scheduled within the 4-week period. t2 mid-point assessments are completed 2 weeks after the start of the intervention. In the post-allocation (t3) stage, participants complete the endpoint assessment online prior to a visit to the Department of Psychology, where they complete the ANT and SART (referred to as cognitive assessment II in Fig. 1). t3 post-assessments are completed 4 weeks after the start of the intervention.

The t4 follow-up takes place 4 months after the start of the intervention. Here, participants in the neurofeedback groups complete assessments online before coming to the Department of Psychology for a booster neurofeedback session. The booster sessions serve the practical goal of probing the participants about their experiences with neurofeedback. Moreover, the visit is included to increase the likelihood of participant adherence to questionnaire completion. For diary control participants, t4 assessments are completed 4 months after the start of the intervention. As a token of appreciation, they then receive a copy of a self-help book on tinnitus [29].

### Randomisation

When, as in our study, specific prognostic factors may influence outcomes, the best way to ensure a balanced distribution of participants across groups is to use an allocation by a minimisation process [30]. Accordingly, participants are stratified to one of the three study groups according to on their age and the severity of their tinnitus [31]. Age is included as a prognostic factor. This is based on the assumption that neurofeedback utilises the brain’s ability to reorganise itself functionally and/or structurally [32, 33]. With the current knowledge, evidence favours the hypothesis that younger individuals have greater neuroplastic potential [34] compared to older individuals. This is partially because of the association between increasing age and functional and structural brain deterioration [35, 36]. However, it would be false to believe that this deterioration affects all individuals to the same degree. With increased age comes large individual differences in brain health [34]. These differences are already apparent by the time people reach their mid-fifties [35]. Taken together, these observations have led to the following stratification categories for age in the present study: 1) ≤ 55 years; 2) > 55 ≤ 64; 3) > 64 ≤ 69; and 4) > 69. Given the conservative inclusion criterion on the primary outcome variable (18 ≥ THI score pre-intervention ≤100), stratifying participants into sub-groups based on the tinnitus distress classification by McCombe and Baguley [31] is considered a sensible option. This ensures a balanced distribution of the range of THI scores across the three groups. Importantly, this reduces the risk of heterogeneity of variance affecting the between-group outcome analysis. Thus, participants are stratified to one of four levels of tinnitus distress: 1) mild (THI 18–36); 2) moderate (THI 38–56); 3) severe (THI 58–76); and 4) catastrophic (THI 78–100).

Allocating participants to one of the three groups is performed by a researcher independent of the study. This individual, trained in the minimisation programme Minim [37], will subsequently inform the research team about the group allocation of each participant. A group allocation list is kept in a separate and password secured Excel file on the local drive of the independent researcher’s PC.

To ensure blinding of neurofeedback trial participants, two identical neurofeedback training protocols were developed. First, information about the course of the training is kept identical for participants in both groups. Second, the position of the training electrodes is the same; that is, positions FC1, FC2, F3 and F4 of the 10/10 International system were used in the training sessions. Third, the duration and intensity of the training sessions are identical for both groups; that is, all participants undergo ten sessions, each initially consisting of four but progressing to five training blocks per session. Fourth, the neurofeedback training stimulus used, i.e., a fish swimming vertically across the screen, is the same for both groups. In other words, all variables are kept constant except for the trained frequency bands, which is information not accessible to participants.

### Interventions

#### Neurofeedback training groups

The neurofeedback training sessions are designed identically for the ADR and the BTR sessions. Both training sessions will be applied unidirectionally, with the aim of a) decreasing the ADR by an increase of alpha [8–12 Hz) and/or a reduction of delta (2–4 Hz) and b) decreasing the BTR by inhibiting theta (4–8 Hz) and/or reinforcing beta (13–20 Hz) activity bilaterally over the fronto-central cortex (FC1, FC2, F3 and F4).

During each neurofeedback (NF) training session, EEG and electromyography (EMG), as well as a vertical and horizontal electrooculogram (EOG), are recorded with Ag/AgCl-sintered ring electrodes with impedances kept below 5 kΩ using a 13-channel DC-amplifier (THERA PRAX®MOBILE, Neurocare GmbH, Ilmenau, Germany; > 10 Gohm input impedance) and a sampling rate of 256 Hz. EEG electrodes are placed at F3, F4, FC1 and FC2 in accordance with the 10/10 electrode placement system [38] using an elastic electrode cap (EasyCap, Woerthsee-Etterschlag, Germany). Reference and ground electrodes are attached to the right and left mastoid, respectively. The electrode montage is identical to previous neurofeedback studies in tinnitus [14, 39] to guarantee comparability with former research. EMG is recorded with two electrodes placed at the upper descending part of the M. trapezius. To reduce artefacts produced by eye movements, a real-time EOG is recorded simultaneously using four electrodes (two electrodes at external canthi, and two electrodes at infra- and supraorbital sides). Eye movements are removed from the feedback signal during the training using an online ocular correction as described by Schlegelmilch and Markert [40]. In addition, all signals acquired during neurofeedback training are stored as raw signals for later offline processing without any calibration or filtering.

**Fig. 2:**
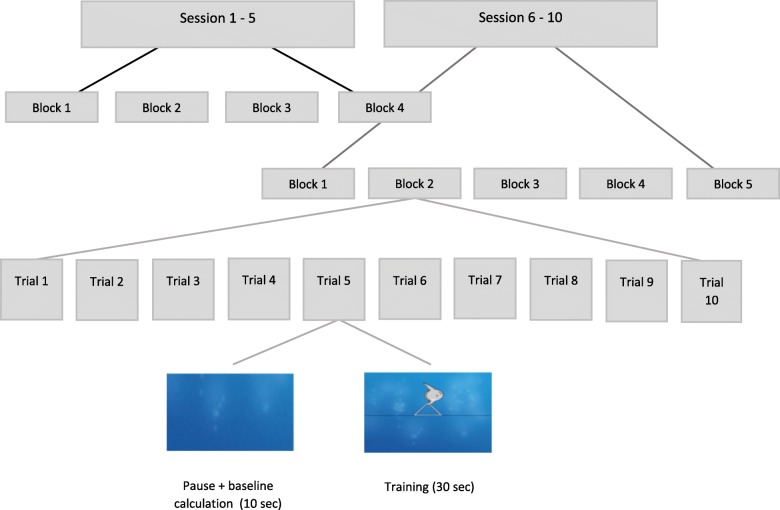
Study flow divided into sessions, training blocks and trials

Online-processing in real time comprises a 50 Hz notch filter applied to EEG signals before direct feedback. Depending on the respective training group, BTR [theta(μV^2^/Hz)-beta(μV^2^/Hz)/theta(μV^2^/Hz) + beta(μV^2^/Hz)] or ADR [delta(μV^2^/Hz)-alpha(μV^2^/Hz)/delta(μV^2^/Hz) + alpha(μV^2^/Hz)] are extracted with a short-time-Fourier transformed moving average across the four training electrodes and fed back to participants’ monitors, using a graphical object (i.e., fish): horizontal movements of the object from left to right at a constant speed represents the temporal proceeding of the trial (i.e., sampling rate), while vertical movements of the object indicates the targeted changes in the feedback parameter (ADR or BTR), either by moving up (targeted change) or down (non-targeted change). A successful change in cortical activity (i.e., keeping the ADR or BTR above the training threshold for at least 250 ms) is rewarded with the symbol of a sun after each trial, as the only performance-dependent reinforcement. The neurofeedback training protocols are largely in line with the successful tau-(8–12 Hz; identical to our alpha frequency band)-to-delta-ratio protocol used previously [13]. It comprises 10 sessions (four runs and 10 trials; net training approximately 30 min) over the course of 4 weeks. One trial consists of 30 s of active neurofeedback training, followed by a 10-s inter trial interval, in which the last 2 s are used to determine the current baseline for both trainings. Therefore, each trial during training is individually baseline-corrected, and the feedback object always starts moving from the same position on the monitor (please see https://youtu.be/0ZD_mUFixHs for an illustration of the individual trial run). The scale of the feedback monitor is set to 2 μV. For the first training trial in the first session, the training threshold is set to 20%, corresponding to reward reinforcement when the ADR or BTR is greater than 0.2 μV for at least 250 ms. If a reinforcement rate of at least 70% is reached for a training block, the threshold is increased by 3%. In blocks with success in less than 30% of the trials, the threshold will be lowered by 2%. The following training session is started with the thresholds of the last block of the preceding session. To enhance transfer effects, we also gradually included more transfer trials in an additional block at the end of each session, starting from session 6 onwards (see Fig. 2). During transfer runs, patients will not receive any continuous feedback, but will receive reinforcement for successful trials (reward symbol; please see https://youtu.be/shcR8Ilq3mo for an example of a transfer trial). The percentage of transfer trials gradually increases with each session (20%, 40%, 60%, 80% and, finally, 100% transfer trials within the final block of a session). The transfer block is implemented to facilitate the transfer of acquired changes into daily life when no feedback of physiological signals can be provided. Transfer trials, the use of which is reviewed comprehensively in [18], have successfully been employed in other frequency-based NF-protocols [41]. To further facilitate transfer, participants are instructed to retrieve their neurofeedback experiences by designing personalised cues (i.e., printed graphics representing the mental strategy used during the neurofeedback training) and to use those cues both during within-session transfer trials and during daily life. After each session, compliance is verified by questioning the participants to identify whether they have used the transfer cards over the intervention period.

Before participating in the first training block in the first session, the participant is shown the training sequence on the monitor. On the trainee monitor, the fish swims horizontally from left to right on the screen in a sequence lasting 30 s. This is followed by a 10 s pause before the process is repeated. During this presentation, the therapist tells the participants that their task is to try to move the fish above the black reference line in the middle of the screen. No further instructions on how to accomplish this is given. This mirrors the instructions given in [13], thus ensuring greater comparability between results. In addition to providing these instructions, during the breaks between training blocks, the therapist briefly discusses the types of mental activities the participants developed and employed to achieve the training goals.

### Diary control group

In the diary control group, Group 3, an eight-item diary is completed each evening for 7 consecutive days, in week one and week three of the 4-week intervention period. The items are purposefully designed to make participants reflect more positively about their experience with tinnitus (e.g., ‘The tinnitus didn’t disturb me today’). As adherence to diary completion is a concern, participants have two supportive face-to-face meetings. In these, participants are informed about the content and structure of the diary and instructed on its completion. In addition, psychoeducation on tinnitus aetiology, the content of which is based mainly on recognised self-help sources [29, 42], is provided. In addition, two follow-up telephone calls are held throughout the 4-week period. The follow-up telephone calls are supportive in nature and give participants an opportunity to share their experience with the diary exercise.

#### Adherence to the interventions

To maximise treatment adherence in clinical trials in general, several suggestions have been proposed (e.g., [43–45]). Of these, three in particular are relevant to our study. First, participants will find it relatively easy to complete the questionnaires in the study because all primary and secondary outcome measures are collected via an easy-to-navigate online platform. For people without access to the internet, pen and paper versions of the questionnaires can be collected at the department or obtained via mail upon specific request. Second, for participants in both the neurofeedback and diary control groups, frequent contact with members of the research team occurs throughout the intervention period. Third, participants in the diary control group are given an incentive—a self-help book [29]—upon completion of all outcome measures.

#### Measures

##### Primary outcome measures

There are two primary outcome measures in the study. First, the change in tinnitus distress following neurofeedback training is measured with the THI [24]. The THI is a 25-item self-report instrument. Each item is rated on a three-point Likert scale with responses of ‘Yes’ = 4 points, ‘Sometimes’ = 2 points, and ‘No’ = 0 points, thus yielding a total score between 0 and 100. The instrument has three subscales, assessing functional, emotional and catastrophic reactions to tinnitus. The overall test-retest reliability of the scale is 0.92 [46]. It has been translated and validated into a German version [47, 48], which is used in the present study.

To assess the efficacy of neurofeedback training in reducing the perceived intensity, i.e., the sound perception of tinnitus, the TMI is used [49]. This is a three-item measure designed to assess the individual perception of tinnitus sound intensity, without overlapping significantly with cognitive, behavioural or emotional reactions to tinnitus. The internal consistency of the scale is excellent, with Cronbach’s α = 0.86; it also has satisfactory discriminant validity (correlation of *r* = .62 with the THI). One obstacle in the development of the TMI, noted by Schmidt and Kerns [49], is the differential scaling of its three items. This has, according to the authors, the potential to increase measurement error. Schmidt and Kerns [49] suggested converting the three items into a standardised scale. Consequently, in the present study, the response scales for all three items are standardised to a range from 0 to 100.

##### Secondary outcome measures

The German version of the Tinnitus Functional Index (TFI) [50, 51] is used in the present study to detect the responsiveness to the intervention. It assesses treatment-related changes in different areas of functionality, e.g. sleep or sense of control. It consists of 25 items, with 23 responses being rated on a Likert scale from 0 to 10, and two items, from 0 to 100. For scoring the TFI, the two items ranging from 0 to 100 are divided by 10, thus yielding a total TFI score of the 25 items between 0 and 100. Eight subscales are associated with the TFI (intrusiveness, sense of control, cognitive interference, sleep, auditory difficulties, relaxation, quality of life and emotional distress). Overall, the TFI has good test-retest reliability (0.78) and convergent (*r* = 0.86 with the THI) and discriminant validity (*r* = 0.56 with Beck Depression Inventory Primary Care) [50].

To assess changes in sleep quality following neurofeedback training, the *Insomnia Severity Index* (ISI) [52] is used. The ISI consists of seven items, with Likert responses ranging from 0 to 4. Thus, a person can score between 0 and 28 on the total scale, with higher values indicating greater sleep disturbances. The ISI has good internal consistency (Cronbach’s α = 0.91, [52]) and convergent validity (r = 0.80 with the Pittsburg Quality Sleep Index, [53]).

Changes in depressive symptoms following neurofeedback are assessed with the Personal Health Questionnaire-9 (PHQ-9 [54];), a nine-item self-report instrument with Likert scale responses ranging from 0 to 3. The total score of the scale thus ranges from 0 to 27. The internal consistency of the PHQ-9 is good (Cronbach’s α = 0.89) and a test-retest reliability of *r* = 0.84 has been demonstrated [55]. Moreover, the PHQ-9 has strong convergent validity with other scores of depression (e.g., the Beck Depression Inventory, *r* = 0.73) and the General Health Questionnaire-12 (*r* = 0.59) [56].

Further secondary exploratory research questions of the current trial focus on the moderating role of pre-treatment expectancy, perceived treatment credibility and somatic self-efficacy on treatment outcome. These moderator analyses are assessed with the following instruments: an adapted form of the treatment credibility and expectancy questionnaire [57], a German version of the somatic self-efficacy questionnaire [58], and an expectancy questionnaire developed specifically for this study.

##### Training outcomes

To keep track of individual learning curves during training, we will analyse the ADR and BTR for each participant within and across sessions. However, session 1 will be discarded because it is assumed that participants will have to habituate to the setting. On the one hand, monitoring and encouraging learning will be performed by the trainers. On a patient monitor, the trainer can keep track of training progression as the percentage decrease in the BTR or ADR compared to the individual baseline for each training block.

On the other hand, the total number of successful trials per training block is used to guide either the upwards or downward adjustment of the training thresholds. Dependent measures include the mean training level over the training blocks within a session (%), the best run of each session (maximum training level) and the total number of obtained rewards per session. Furthermore, electrophysiological training raw data gathered with the THERA PRAX® MOBILE described previously will be analysed offline with Brain Vision Analyzer v2.0 (Brain Products GmbH, Gilching, Germany).

*Preprocessing:* Data are first band-pass filtered with Butterworth zero-phase filters between 0.1 Hz and 80 Hz with slopes of 24 dB/octave at the low and 48 dB/octave at the high cut-offs. To eliminate possible line noise, the data are further refined using a band-rejection filter with a central frequency of 50 Hz, a bandwidth of 1 Hz, and a slope of 24 dB/octave. For ocular correction, we will use Gratton and Coles’ [59] algorithm, as implemented in Brain Vision Analyzer software. Using a semi-automatic raw data inspection procedure, the recorded data from the training blocks will be screened for artifacts. Planned criteria for artifact screening will be as follows: maximal voltage step of 50 μV/ms, maximal amplitude of ±100 μV, maximum allowed difference of 150 μV in each segment, values greater than 200 μV per 200 ms interval and activity below 0.5 μV in a 50-ms period as the criteria. A total of 100 ms of data will be removed before and after any detected artifacts. A thorough visual inspection will be performed to remove any possible remaining artifacts (i.e., muscle movements and short drifts or jumps over single or multiple electrodes) from the signal.

*EEG analysis:* The artifact-free training data will be segmented into non-overlapping 2-s epochs and submitted to a fast Fourier transformation (FFT) with a 10% Hamming window. The resulting data are averaged in the frequency domains (delta 2–4 Hz; theta 4–8 Hz; alpha 8–12 Hz and beta 13–20 Hz) for feedback versus transfer trials for each individual session and each individual patient across the four training electrodes. Absolute power values (μV2) for each frequency band (i.e., delta, theta, alpha and beta), theta/beta and delta/alpha power ratios, and relative power (%) are calculated.

#### Data management and monitoring

Upon registration on the web platform, participants are assigned a computer-generated study code, which will follow them throughout the trial. All collected data are stored and pseudonymised in locked cabinets or as computer files. The pseudonymisation allocation list is kept separate from the pseudonymised data and deleted upon completion of the trial. Participants will be informed of this procedure. Only people who have signed a confidentiality agreement and are part of the research team have access to the data collected in the study.

The study’s assessment questionnaires are answered online. All necessary precautions have been taken to ensure data protection and security. All data exchanged between participants and the online system are encrypted prior to transmission and storage. The online system is managed and hosted by the IT Department of Linköping University (Sweden). No entries in the form of video or sound recordings, which could make it possible for third parties to identify the participants, are registered. Until the pseudonymisation allocation list is deleted, participants can ask for their data to be deleted at any time upon stating their assigned code. Lastly, data collected during the study are kept for 10 years before being deleted.

### Adverse events

Neurofeedback is generally considered safe and involves no risk to participants [60]. This is perhaps best reflected in the fact that it has been used extensively in studies involving children with ADHD. Thus, participants in our trial should not experience more serious side effects than perhaps mild headaches, stemming mainly from the prolonged period of sustaining attention during training. Nevertheless, the adverse effects of neurofeedback training will be routinely monitored as part of the ongoing dialogue between data collectors and trial participants. In the unlikely event that a trial participant complains about serious adverse effects as a result of the training, data collectors will, as a first step, reduce the frequency of training sessions in the week of the complaint and, if needed, reduce the number of training segments per training session until the trial participant no longer complains about adverse effects.

### Ethics and dissemination

Should any future modification to the protocol occur that changes the study objective, design or procedures, this change will be addressed by the study’s principal investigator. Substantial protocol amendments are submitted to and approved by the responsible ethics committee, prior to implementation.

Dissemination of results is expected to follow the completion of data collection, scheduled to last until late spring in 2020. The results of the primary outcome measures are reported and disseminated, regardless of the direction and magnitude of effect(s). No restrictions are imposed on which results can be disseminated from either the trial sponsor or other interested parties.

### Statistical analysis

Data will be analysed primarily in the intention-to-treat (mITT) population. Supportive analyses are planned in the per-protocol (PP) population. mITT comprises all randomised patients, while PP analysis assesses mITT patients who show no change in their status during the trial period that violates the criteria for their inclusion/exclusion (for example, the data from someone seeking another type of treatment for their tinnitus during the trial period is excluded from further analyses).In addition, data from participants who deviate significantly from the visit schedule are excluded from analyses, and data from participants who show poor compliance during feedback sessions are excluded (e.g., showing sleepiness or lack of focus and orientation to the task). For all outcome measures, we will probe the longitudinal course across all assessments using a linear mixed model for repeated measures (MMRM, see [61]). The MMRM model includes fixed effects for group (ADR vs. TBR vs. diary control), time and group-by-time interaction, as well as a random intercept for subject specific effects using maximum likelihood estimation, adding sex, age, baseline tinnitus severity scores and patients’ expectations as covariates. The error variance-covariance matrices for the repeated factor will be specified in accordance with the data. The pattern of missing data is assumed to be random.

## Discussion

The present study was developed with the aim of investigating not only the specificity of a neurofeedback training protocol but also to identify non-specific effects and their relative contribution to treatment outcomes. Non-specific effects are suggested as being putative healing mechanisms in neurofeedback [16] and their influence in clinical trials have not been sufficiently taken into account in previous studies, where appropriate control groups were missing. In keeping all variables constant between the two neurofeedback groups (ADR vs. BTR), except for the trained frequency bands, our study is designed in such a way that only the components under investigation are manipulated.

An alternative design with different components for the neurofeedback control group could have been implemented instead of the chosen, almost identical BTR group. It is vital to acknowledge that any control group chosen in this study, as in general (e.g., see [62, 63]), comes with its own set of advantages and disadvantages. Deciding upon control conditions ultimately comes down to considerations of the advantages and disadvantages of different types of control conditions. For this study, a wait-list control group seems like the least attractive option. This type of control tends to overestimate the effects of the intervention under examination [64]. Sham or placebo neurofeedback was also considered but not selected. Important considerations included, first, that when alternative treatment options exist, this type of design should be avoided [65]; secondly, placebo or sham neurofeedback may affect participant motivation and expectancy in a negative manner [66, 67]; and third, for the same reasoning as in [9], sham neurofeedback is unjustified in a study in which participants are expected to invest considerable time without receiving monetary compensation.

The decision to include a diary control group was informed by the criticism that the treatment effects of neurofeedback appear less attributable to the self-regulation of brain activity per se, but to a greater extent, might be driven by non-specific factors such as, for example, heightened expectations [68]. A common method of controlling for non-specific effects in intervention studies is to include a non-component control group [62]. A characteristic of this is the inclusion of elements that control for attention and outcome expectancy whilst leaving out the supposedly active intervention component (in the present study, neurofeedback). The consultations and telephone follow-up calls that are implemented as part of the protocol for the diary participants help to establish a client/therapist relationship. The psychoeducational element is implemented to provide a framework in which participants can understand how the daily completion of a diary, in addition to doing the recommended exercises, can facilitate increased well-being. Providing this treatment rationale should ultimately promote greater optimism, which in turn may positively affect outcome expectancy. In short, the purpose of the three-arm design in the present study is two-fold. It allows us to 1) assess the efficacy of a specific neurofeedback training protocol (ADR vs. BTR) and 2) investigate the added non-specific effects of neurofeedback training (ADR + BTR vs. diary control).

Although double-blinding is considered the gold standard in randomised controlled trials, this study is non-blinded. Some proponents of neurofeedback argue that double-blinding in neurofeedback poses a significant challenge [69] and that the field is not yet mature enough to embrace the double-blind design [70]. The main technical challenge in the double-blind design in neurofeedback is that it necessitates the use of an automatic threshold setting in training. Thresholds in neurofeedback are used to reward behaviour and thereby shape it in the desired direction. When automatic threshold procedures are used, reward thresholds are adjusted moment by moment to ensure rewards for a certain percentage of time based on the previous averaged period, e.g., 15 s. However, if overall performance deteriorates, reward thresholds are adjusted downwards to adapt to the new output level. In doing so, despite a deterioration in performance, rewards are still elicited. The consequence of this is that behavioural shaping, the crucial goal in neurofeedback [69], is hampered. When this happens, learning processes are impaired [18, 69, 71, 72]. As a result, given this consideration whilst also acknowledging the absence of universal consensus on the matter, we opted for a design in this study in which only participants undergoing neurofeedback are blinded to their intervention.

The decision to use a between- rather than within-subject design was made only after several discussions and reflections. In the end, a between-subjects design was decided upon for two reasons. First, participation in this study requires a considerable time investment. In the within-subjects design, participants would have to set aside at least 8 weeks, spaced over two 4-week periods, with two to three training visits to the department per week. This factor, we feared, would increase the risk of participant drop out. Second, when the effects of a condition are expected to be persistent, a risk of carrying it over to the second condition exists, which may confound the outcomes [73]. Therefore, although this type of study requires a larger number of participants, we opted for the between-subjects design.

### Limitations

The intent-to-treat statistical method applied in the study is particularly vulnerable in relation to treatment adherence and drop-out. The willingness of participants to complete all outcome measures, independent of their status in the trial, is crucial to avoid the distortion of study results. To maximise treatment adherence and minimise drop-out in the present study, based on recommendations in the literature (e.g., [43–45]), several steps have been taken to minimise the risk.

The non-blinding of therapists in the neurofeedback conditions mean the outcomes are more susceptible to the experimenter effect. This term covers situations in which the experimenter, with awareness of the components of interventions, unconsciously or unwittingly behaves in ways towards participants that promote the favoured hypotheses [74]. To address this as best we could, a protocol containing standardised verbal instructions has been developed and used. Therefore, although the experimenter effect cannot be eliminated in its entirety, we hope this strategy, at best, minimises its impact. Regardless, the randomised controlled study design without blinding has its pitfalls, potentially influencing outcomes. Therefore, it must be considered a limitation in the study.

In conclusion, with our study we hope to take the first steps in understanding factors that promote and/or underpin reported health benefits in tinnitus populations who undergo neurofeedback treatment.

## Trial status

Issue date: 17 March 2020. Protocol version no. 3.

Recruitment commenced on 1 November 2018 and is expected to be completed in June 2020.

## Supplementary information

**Additional file 1.** SPIRIT 2013 Checklist: Recommended items to address in a clinical trial protocol and related documents.

**Additional file 2.** Response to the addendum to the “Neurofeedback for tinnitus” application to the Ethics Committee of the Department of Psychology (file number 2018-04k).

**Additional file 3.** Study: “Neurofeedback for tinnitus - does frequency band specificity matter?”.

**Additional file 4.** Information about the study "Tinnitus and Neurofeedback" (ToNe-study).

**Additional file 5.** Probandeninformation zur HNO-ärztlichen Untersuchung im Rahmen der Studie. “Tinnitus und Neurofeedback“ (ToNe-Studie).

**Additional file 6.** Participant Information regarding the ENT assessment part of the study "Tinnitus and Neurofeedback" (ToNe study).

**Additional file 7.** Trial registration—dataset.

## Data Availability

De-identified, limited data will be made available upon reasonable request from the corresponding author.

## Abbreviations

ADHD: attention deficit hyperactivity disorder
ADR: alpha/delta ratio
ANOVA: analysis of variance
ANT: attention network task
BTR: beta/theta ratio
EEG: electroencephalography
EMG: electromyography
ENT: ear-nose-throat
EOG: electrooculogram
FFT: fast Fourier transformation
Hz: Hertz
ISI: insomnia severity index
MANOVA: multivariate analysis of variance
mITT: intention-to-treat
MMRM: mixed model for repeated measures
OAE: otoacoustic emission
PP: per-protocol
PHQ-9: Personal Health Questionnaire-9
SART: sustained attention to response task
STAI: State Anxiety Inventory
TFI: Tinnitus Functional Index
THI: Tinnitus Handicap Inventory
TMI: Tinnitus Magnitude Index
NF: neurofeedback
