# Supplementary material for: Neurofeedback for tinnitus: study protocol for a randomised controlled trial assessing the specificity of an alpha/delta neurofeedback training protocol in alleviating both sound perception and psychological distress in a cohort of chronic tinnitus sufferers
Source: Trials. 2020 May 5;21:382. doi: 10.1186/s13063-020-04309-y (PMC7201543; doi:10.1186/s13063-020-04309-y)
Supplement: Supplementary file 2 — Additional file 2. Response to the addendum to the “Neurofeedback for tinnitus” application to the Ethics Committee of the Department of Psychology (file number 2018-04k). [file 13063_2020_4309_MOESM2_ESM.pdf]

Philipps Universität Marburg

Fachbereich Psychologie
Dr. Cornelia Weise

- im Hause -

Fachbereich Psychologie
AE Entwicklungspsychologie
Prof. Dr. Martin Pinquart
Vorsitzender der Ethikkommission
Tel.: 06421 – 2823626
Fax: 06421 – 2823685
E-mail: pinquart@uni-marburg.de
Anschrift: Gutenbergstraße 18
35032 Marburg

Marburg, den 06.06.2018

Stellungnahme zum Addendum zum Antrag „Neurofeedback bei Tinnitus“ an die Ethik-Kommission des Fachbereichs Psychologie (Aktenzeichen 2018-04k)

Sehr geehrte Frau Weise,

gegen die im Addendum genannten Modifikationen Ihres Antrags bestehen keine grundlegenden ethischen Bedenken.

Zudem weist die Kommission explizit darauf hin, dass ihr Votum in einer Empfehlung besteht, die weder die Prüfung von Datenschutzrecht zum Gegenstand hat, noch den Zweck und das Ziel, die antragstellenden Wissenschaftlerinnen und Wissenschaftler von der Notwendigkeit der Einhaltung des geltenden Rechts zu entlasten. Zudem empfiehlt die Kommission eine Beratung durch den lokalen Datenschutzbeauftragten der Universität in Hinblick darauf, ob die Datenerhebung und Datenspeicherung, sowie die Teilnehmerinformationen und Einwilligungen der in Kraft getretenen EU-Datenschutz-Grundverordnung genügen.

Die Ethik-Kommission (EK) übernimmt keinerlei Haftung für Schäden, die aus einer wissenschaftlichen Untersuchung entstehen, zu der die EK auf Antrag Stellung genommen hat. Dieser Haftungsausschluss gilt auch für den Fall, dass sich der Antragsteller bei der Untersuchungsdurchführung an die Empfehlungen der EK hält, es sei denn, die EK hat vorsätzlich oder grob fahrlässig fehlerhafte oder rechtswidrige Empfehlungen abgegeben.

Eine erneute Vorlage des Antrags und der Unterlagen ist nicht erforderlich.

Mit freundlichen Grüßen

Pinquart

Mit freundlichen Grüßen
