# Supplementary material for: Neurofeedback for tinnitus: study protocol for a randomised controlled trial assessing the specificity of an alpha/delta neurofeedback training protocol in alleviating both sound perception and psychological distress in a cohort of chronic tinnitus sufferers
Source: Trials. 2020 May 5;21:382. doi: 10.1186/s13063-020-04309-y (PMC7201543; doi:10.1186/s13063-020-04309-y)
Supplement: Supplementary file 3 — Additional file 3. Study: “Neurofeedback for tinnitus - does frequency band specificity matter?”. [file 13063_2020_4309_MOESM3_ESM.pdf]

Philipps Universität Marburg

Philipps-Universität - 35032 Marburg

Frau Dr. Cornelia Weise
Philipps-Universität Marburg
FB Psychologie
AG Klinische Psychologie u. Psychotherapie
Gutenbergstraße 18
35032 Marburg

Herrn Prof. Dr. Boris A Stuck
Universitätsklinikum Gießen u. Marburg GmbH
Standort Marburg, Klinik für Hals-Nasen-Ohrenheilkunde
Baldingerstraße
35033 Marburg

Fachbereich Medizin
Dekanat/Ethikkommission
Prof. Dr. med. Gerd Richter (Vors.)

Tel.: 06421 586 6487
Fax: 06421 586 6585
Sek.: D. Raiss/S. Hausmann
E-Mail: ethikkom@staff.uni-marburg.de
Anschrift: Baldingerstrasse/Postfach 2360
35032 Marburg
Web: www.med.uni-marburg.de/ethikkomm
Az.: 162/18

Marburg, den 02.01.2019

Studie: „Neurofeedback for tinnitus – does frequency band specificity matter?".

Eingereichte Unterlagen (Eingang 13.11.2018):

1. Anschreiben vom 12.11.2018
2. Ethikantrag vom 12.11.2018
3. Probandeninformation
4. Einwilligungserklärung der Probandin, des Probanden
5. Positive Voten der Ethikkommission FB Psychologie der Philipps-Universität Marburg vom 09.02.2018 und 06.06.2018
6. Addendum Ethikantrag „Neurofeedback bei Tinnitus" (Aktenzeichen 2018-4k) vom 15.05.2018
7. Information für Teilnehmerinnen und Teilnehmer der Studie „Neurofeedback bei Tinnitus" (ToNE-Studie)
8. Einverständniserklärung zur Teilnahme an der Studie Tinnitus und Neurofeedback „ToNE-Studie"
9. Information für Teilnehmerinnen und Teilnehmer der EEG-Studie „Gehirnaktivität bei Gesunden und Tinnitusbetroffenen"
10. Einwilligungserklärung zur Teilnahme an der EEG-Studie „Gehirnaktivität bei Gesunden und Tinnitusbetroffenen"
11. Appendix
12. Studienflyer

Unterlagen (Eingang 27.11.2018):

1. Brief von Frau Dr. C. Weise und Prof. Dr. B. Stuck vom 26.11.2018
2. Anschreiben vom 27.11.2018
3. Universität-interne Kooperationsvereinbarung
4. Ethikantrag gemäß Marburger Checkliste (keine Versions-Nr.), datiert vom 26.121.2018
5. Probandeninformation zur Studie „Neurofeedback for tinnitus – does frequency band specificty matter?" (HNO-Teil)
6. Einwilligungserklärung der Probandin, des Probanden (HNO-Teil)
7. Ethics application for the study: Neurofeedback for tinnitus – does frequency band specifity matter? (keine Versions-Nr., kein Versions-Datum)
8. Information für Teilnehmerinnen und Teilnehmer der Studie „Neurofeedback bei Tinnitus" (ToNE-Studie) (keine Versions-Nr., Kein Versions-Datum)
9. Einverständniserklärung Teilnahme an der Studie Tinnitus und Neurofeedback: „ToNE-Studie" (kein Versions-Datum, keine Versions-Nr.)
10. Information für Teilnehmerinnen und Teilnehmer der EEG-Studie „Gehirnaktivität bei Gesunden und Tinnitusbetroffenen" (Studie im Rahmen der ToNE-Studie) (keine Versions-Angabe, kein Versionsdatum)
11. Einwilligungserklärung zur Teilnahme an der EEG-Studie „Gehirnaktivität bei Gesunden und Tinnitusbetroffenen" (keine Versions-Angabe, kein Versions-Datum)

Kommissionsmitglieder: □ Prof. Dr. Jens Puschke, □ Prof. Dr. med. J.-C. Krieg, □ Prof. Dr.med. Czubayko, □ Prof. Dr. med. G. Richter (Vorsitzender), □ PD Dr. Carola Seifart, □ Prof. Dr. med. Uwe Wagner (stellvertretender Vorsitzender), □ Prof. Dr. med. R. Maier, □ Prof. Dr. med. N. Donner-Banzhoff, □ Dr. rer. nat. Nina Timmesfeld, □ Prof. Dr. med. A. Neubauer, □ PD Dr. B. Tackenberg, □ PD Dr. Barbara Fritz □ Bettina Nieth, □ Dr. Thomas Neubert

12. Agreement on the Transfer of granted Funds
13. Information zur Studie „Neurofeedback bei Tinnitus" (ToNE-Studie) (keine Versionsangabe, kein Versionsdatum)
14. Einverständniserklärung zur Teilnahme an der Studie „Tinnitus und Neurofeedback": ToNE-Studie (keine Versionsangabe, kein Versionsdatum)
15. Patienteninformation gemäß DS-GVO, Versionsdatum 07.06.2018
16. Fragenbogenbatterie
17. 2 Flyer zur Rekrutierung

Unterlagen (Eingang 21.12.2018):

1. Ihre Stellungnahme vom 20.12.2018 zum Gutachten der Ethikkommission vom 03.12.2018
2. Studieninformation & Einverständniserklärung zur ToNe-Studie Version 2018-01 vom 17.12.2018
3. Information zur Datenschutz-Grundverordnung ToNe-Studie DSGVO_v.2018-01 vom 17.12.2018
4. Studieninformation & Einverständniserklärung zur HNO-ärztlichen Untersuchung im Rahmen der ToNe-Studie HNO_v. 2018-01 vom 17.12.2018

Sehr geehrte Frau Dr. Weise,
sehr geehrter Herr Professor Stuck,

vielen Dank für Ihre Stellungnahme vom 20.12.2018 zu unserem Gutachten vom 03.12.2018. Die von uns aufgeworfenen Fragen wurden alle ausreichend beantwortet und entsprechende Modifikationen bzw. Korrekturen eingefügt, so dass nunmehr keine berufsethischen und berufsrechtlichen Bedenken gegen die Studie vorliegen.

Damit schließt die Ethikkommission des Fachbereichs Medizin der Philipps-Universität Marburg ihr Gutachten mit einem

positiven Ethikkommissionsvotum

ab.

Entsprechend der ausschließlich beratenden Funktion der Ethik-Kommission betrifft dieses Votum nur die ethische Beurteilung der Konzeption, der vorgesehenen Methoden, der Durchführung und Überwachung des betreffenden Projektes sowie der beabsichtigten Patientenaufklärung. Die ärztliche und juristische Verantwortung verbleibt jedoch uneingeschränkt beim Projektleiter und seinen Mitarbeitern.

Bitte geben Sie uns jede Änderung in der Protokolldurchführung bekannt. Es muss dann geklärt werden, ob das Votum der Ethik-Kommission noch Gültigkeit hat. Bitte berücksichtigen Sie, dass nachgereichte Unterlagen mit einer Versionsnummer und einem Versionsdatum zu versehen sind (Fußzeile), um deren Identifizierbarkeit bei der Votierung zu ermöglichen. Bitte berücksichtigen Sie ferner, dass eine Bearbeitung nur möglich ist, wenn sämtliche Unterlagen als einzelne Dokumente, auch in elektronischer Form (etwa auf CD) eingereicht werden.

Über alle schwerwiegenden oder unerwarteten unerwünschten Ereignisse, die während der Studie auftreten und die Sicherheit der Studienteilnehmer oder die Durchführung der Studie beeinträchtigen könnten, muss der Vorsitzende der Ethik-Kommission unterrichtet werden.

<u>Hinweise zu den datenschutzrechtlichen Aspekten:</u>
Details zu Ihren Informationspflichten gegenüber Studienteilnehmern entnehmen Sie bitte insbesondere Art. 13 ff DS-GVO. Die Ethikkommission prüft die Angaben zu den zuständigen Datenschutzbeauftragten und Aufsichtsbehörden nicht auf Richtigkeit.
Datenschutzrechtliche Aspekte von Forschungsvorhaben werden durch die Ethikkommission grundsätzlich nur kursorisch geprüft. Dieses Votum / diese Bewertung ersetzt mithin nicht die Konsultation des zuständigen Datenschutzbeauftragten.

Die Ethik-Kommission des Fachbereichs Humanmedizin der Philipps-Universität Marburg arbeitet gemäß den nationalen gesetzlichen Bestimmungen und den ICH-GCP-Richtlinien.

Außerdem benötigt die Ethik-Kommission einen Bericht nach Abschluss der Studie.

Mit freundlichen kollegialen Grüßen
für die Ethik-Kommission des
Fachbereichs Humanmedizin
der Philipps-Universität Marburg

Prof. Dr. med. G. Richter
(Vorsitzender Ethikkommission)
