# Supplementary material for: Neurofeedback for tinnitus: study protocol for a randomised controlled trial assessing the specificity of an alpha/delta neurofeedback training protocol in alleviating both sound perception and psychological distress in a cohort of chronic tinnitus sufferers
Source: Trials. 2020 May 5;21:382. doi: 10.1186/s13063-020-04309-y (PMC7201543; doi:10.1186/s13063-020-04309-y)
Supplement: Supplementary file 4 — Additional file 4. Information about the study "Tinnitus and Neurofeedback" (ToNe-study). [file 13063_2020_4309_MOESM4_ESM.pdf]

Principal investigator: Dr. C. Weise
Philipps-Universität Marburg
FB Psychologie | Klinische Psychologie
Gutenbergstraße 18 | 35032 Marburg
tinnitus@uni-marburg.de

Information about the study "Tinnitus and Neurofeedback" (ToNe-study)

Chronic subjective tinnitus – a multitude of treatment options?

It rushes, it whistles, it buzzes – you know it just too well? Tinnitus is a very common condition in the population. Even if most affected individuals adapt reasonably well to the tinnitus with time, a significant proportion of the affected persons experience it as a very burdensome condition. Tinnitus is affecting the quality of life and it can cause feelings of despair, fear or concentration problems.

Even though there is a multitude of treatments, there is not yet a method, which can eliminate tinnitus. Thus, the primary treatment goal, e.g. with Cognitive Behavioral Therapy, is to reduce the distress caused by the ear noises and thereby enhance the quality of life. However, studies show that a significant proportion of tinnitus patients do not benefit from this kind of psychotherapy. Therefore, it is the aim to develop new treatment alternatives and to analyze their effectiveness.

The aim of our study: neurofeedback – a new treatment alternative?

Recently, neuroscience has become more important in our understanding of tinnitus.
Studies show that changes in brain wave activity can contribute to the development and perpetuation of tinnitus. Brain waves are comprised of the electrical discharge of groups of brain cells, which can be measured with the aid of electroencephalography (EEG) on the surface of the head. The frequency of the brain waves is different, depending on the state we are in: when we are awake and concentrated the brain waves are small, quick and regular and when we sleep deeply, they are slow and big. In tinnitus, the pattern of brain waves is possible disturbed.
Neurofeedback (NFB) is a method that is based on EEG: the measured brain waves get fed back to a monitor and are therefore made “visible”. NFB is believed to help individuals affected by tinnitus change their unhealthy brain activity towards a more desired or healthy state. But at present, there are only a few findings about which brain waves should be trained to get an effect.
And this is the problem the ToNe study adresses: We want to investigate, whether neurofeedback is effective in reducing the loudness of tinnitus and the distress caused by it, and if it makes a difference, which brain waves are trained. To investigate our research question, we conduct a so-called “randomized-controlled study”. This means, that all participants get randomly assigned to one of three groups, either to one of two forms of neurofeedback or to a diary-intervention. At the end of the study, we hope to be able to address the question, whether there are differences between the treatment options.

Requirements for the participations in the study

The ToNe-study is aimed at people, who have had Tinnitus for at least six months (chronic tinnitus), and who feel considerably affected by it. Participants should be motivated to take part in the treatment and have enough time for the participation in the study (filling out questionnaires/ diaries; participation in up to 3 appointments per week over a period of 5 weeks at the Faculty of Psychology in Marburg).

Procedure of the study

If you are interested in participating in the study, you can inform yourself in greater detail about the study on the homepage of the study http://tone.iterapi.se,where you can complete a self-

Principal investigator: Dr. C. Weise
Philipps-Universität Marburg
FB Psychologie | Klinische Psychologie
Gutenbergstraße 18 | 35032 Marburg
tinnitus@uni-marburg.de

test to assess, if a participation is relevant for you. After that, you can register for the study with your e-mail-address and a self-chosen password. Since study registration and completion of the questionnaires is done online, easy access to the internet is desirable[1]. After the registration you will receive an e-mail with a link to a *screening-questionnaire,* which we use to assess, if you are eligible for the trial or not. At the very beginning of the questionnaire, you will be asked to declare your consent to completion of that questionnaire. If you are eligible for further trial participation, we will agree on a date for a *phone interview*, in which we will talk about further relevant information for study participation and clarify open questions. In addition, we will set up a date for a first diagnostic session at the faculty.

At the beginning of *the diagnostic session*, you will be more thoroughly informed about the study. If you agree to further participation, we require you to sign a written consent.
In addition to that, you will be given access to a detailed *online questionnaire,* which you can complete at home. Then, the *first psychophysiological measurement* takes place. With the help of an EEG-device, we will measure your brain activity, in an active and in a resting state. For this purpose, electrodes will be attached to your head with the help of an elastic cap. The EEG recording is not associated with any risks. The connection between the electrodes and the scalp is made with the help of an electrode gel. The applicated paste is clinically tested and easy to wash out after the session. In rare cases, skin irritation or pressure marks are possible at the position where the electrodes were placed. Please tell us if you have skin allergies or a skin oversensitivity.

In a third step of the diagnostic process we will ask you to go to an appointment to the Ear Nose and Throat Department at the University Hospital Marburg. There, an *ENT-examination* and a measurement of your hearing ability will take place. If there are no findings that speak against the participation in the study, we will include you in the study and assign you randomly to one the three groups: (1) Neurofeedback-training-1, (2) Neurofeedback-training-2, (3) Tinnitus diary intervention.

If you get randomly assigned to one of the two neurofeedback groups (NFB-1 or NFB-2) you take part in 10 sessions (5 weeks in total) of neurofeedback training at our faculty. After each neurofeedback training session, you will fill out a very short questionnaire, where you can evaluate your success of the just completed session. If you get assigned to the diary group, you will first have a personal consultation at the faculty. There, you will get information about the diary intervention and receive the tinnitus diary. You must complete the diary twice, for 7 consecutive days each time, in a total treatment period of four weeks. Additionally, you will be contacted weekly via phone to discuss your experiences with filling in the diary and to clarify possible questions. The diary intervention is about observing your tinnitus and its possible association with stress, enabling you to develop strategies to help you cope better with tinnitus long-term.

When about half of the neurofeedback training or the diary intervention is over, you will get an e-mail with a link to a short *interim questionnaire*. After the completion of the training or the diary intervention (approx. 5 weeks after the allocation to the groups), we will send you a link to a more detailed *study questionnaire*. Additionally, we will set up an appointment for the *second psychophysiological measurement* where another EEG will be conducted.

Three months after the end of the neurofeedback training or the diary intervention (that means 5 months after the beginning of the study), we will contact you via e-mail and ask you to complete the *final questionnaire*. In addition, we will invite you to a *third psychophysiological measurement* at our faculty. This is an opportunity for you to refresh what you have learnt and get further insights in your brain activity. These final measurements are very important for us to evaluate the short- and long-term effects of the different treatment approaches. At the end, participants of the diary intervention group get offered an evidenced-based self-help book about coping with Tinnitus, which is based on cognitive-behavioral therapeutic exercises and aims at extending tinnitus coping competences.

[1] If you do not have a computer/ internet access we will provide printed documents for you.

Data privacy and voluntariness

We explicitly point out that participation in the study cannot replace regular psychotherapy or medical treatment. Participation in the study is voluntary. There is no financial compensation for the participation in the study. If, during the course of the intervention, you choose not to continue your participation, you can revoke your consent at any time and without stating reasons. This will not bring any disadvantages for any further or future treatment options. According to the rules of data protection and confidentiality (Federal Data Protection Act §40), information about you are stored in a strictly pseudonymous form. This means, that all collected data are assigned a personal code and stored under that code. An allocation list, in which your name is combined with your unique study code is created. This allocation list is stored in a separate file location, and only accessible to the research team, who have signed a confidentiality agreement. After completion of the data collection (latest on 31.07.2021), the allocation key will be deleted. Thus, your data will be available in anonymous form only. From then on, the data are in full anonymized form, i.e. it is no longer possible to associate personal information with the collected data. Based on legal regulations, the anonymized data are stored for 10 years. As long as the allocation list exists, that is until the completion of the data collection, you can ask for the deletion of your collected data at any time. Your data will be used exclusively for the purposes of the study. The anonymous results of the study can be published as scientific publications. All project employees are subject to confidentiality. All collected data is encrypted and transmitted over a secure connection. All necessary precautions have been taken to ensure data protection and data security. All communication by e-mail is stored encrypted. Our online system and all data collected (registration, questionnaires) are stored on servers provided and managed by the IT department of Linköping University (Sweden).

This study information is part of the informed consent to participate in the study "Tinnitus and Neurofeedback". If you have any further questions about the study or the procedure of the study or if you have any questions during your participation, you are welcome to contact us (e-mail: tinnitus@uni-marburg.de or Tel.: +49 (0) 6421/2823794)

The team of the ToNe-study

Dr. Cornelia Weise (principal investigator)
Martin Jensen, M.Sc & Eva Hüttenrauch, M.Sc. (study assistants)

Dr. Cornelia Weise
– ToNe-Studie –
Philipps-Universität Marburg
Fachbereich Psychologie
Gutenbergstr. 18

35032 Marburg

Fachbereich Psychologie

AG Klinische Psychologie & Psychotherapie

Dr. Dipl.-Psych. Cornelia Weise

Tel.: 06421 2823794
E-Mail: tinnitus@uni-marburg.de

Anschrift: Gutenbergstraße 18
35032 Marburg

Informed consent
for the participation in the study "Tinnitus and Neurofeedback" (ToNe-study)

I have been fully informed about the content and the procedure of the ToNe study, which is investigating the effectiveness of neurofeedback training in reducing tinnitus distress. I agree to fill out the questionnaires related to the study participation and to participate in the neurofeedback training or the diary intervention, depending on which group I am assigned to. If I had questions about this study, they were completely answered by the study team. I agree with the described collection and processing of the data. The recording and evaluation of this data is pseudonymized, i.e. using a number and without stating my name. There is an electronic coding list, which connects my name with this number. This coding list is only accessible to the principal investigator and the researchers in the study. The coding list is locked and stored separately from all other collected data; third parties have no access. After completing the data collection (latest on 31.07.2021), the coding list will be destroyed. Then, my data is only available in anonymous form and can no longer be associated with my name. I am informed that I can revoke my consent to the storage of my data without this leading to any disadvantages for future therapy/treatment. I can request a deletion of all my data as long as the coding list is available.

I agree that my completely anonymized data will be used for research purposes. For this purpose, they are kept at least 10 years after data evaluation, or at least 10 years after a publication of this study.

I had enough time for a decision to be made and I am ready to participate in the above-mentioned study. I know that participating in the study is completely voluntary and not remunerated. I can terminate the participation at any time without giving reasons.

I have received a copy of the study information and a copy of the consent form. The study information is part of this declaration of consent.

**Surname, name:**									
**Study code** (which you got from us, e.g. dyfd0031)									

Place and date	Signature participant	Signature principal investigator
