# Supplementary material for: Neurofeedback for tinnitus: study protocol for a randomised controlled trial assessing the specificity of an alpha/delta neurofeedback training protocol in alleviating both sound perception and psychological distress in a cohort of chronic tinnitus sufferers
Source: Trials. 2020 May 5;21:382. doi: 10.1186/s13063-020-04309-y (PMC7201543; doi:10.1186/s13063-020-04309-y)
Supplement: Supplementary file 5 — Additional file 5. Probandeninformation zur HNO-ärztlichen Untersuchung im Rahmen der Studie. “Tinnitus und Neurofeedback“ (ToNe-Studie). [file 13063_2020_4309_MOESM5_ESM.pdf]

UNIVERSITÄTSKLINIKUM GIESSEN UND MARBURG

Universitätsklinikum Gießen und Marburg GmbH • Klinik für HNO-Heilkunde
Baldingerstr., 35043 Marburg

STANDORT MARBURG
Klinik für Hals-, Nasen- und Ohrenheilkunde

Direktor:
Prof. Dr. Boris A. Stuck
Baldingerstr.
35043 Marburg
Telefon: 06421-5866478
Telefax: 06421-5866367
Mail:sekretariat.hno.mr@uk-gm.de
Internet: www.ukgm.de
Datum:
Zeichen: stuck-

Probandeninformation zur HNO-ärztlichen Untersuchung im Rahmen der Studie „Tinnitus und Neurofeedback“ (ToNe-Studie)

Sehr geehrte Probandin, sehr geehrter Proband,
hiermit möchten wir Sie über die oben genannte Studie informieren:

Bezüglich der grundsätzlichen Hintergründe, Fragestellungen und methodischen Ansätze der Studie verweisen wir auf die Aufklärungsbögen des Fachbereichs Psychologie, AG Klinische Psychologie und Psychotherapie, Philipps-Universität Marburg, den Sie bereits erhalten haben.

Im Rahmen der Studie ist es erforderlich, dass wir bei Ihnen eine Otoskopie und ggf. eine Gehörgangsreinigung durchführen. Hierbei wird HNO-ärztlich in Ihre Gehörgänge gesehen und ggf. sanft eventuell vorhandenes Cerumen („Ohrenschmalz“) entfernt. Dies ist weder invasiv noch schmerzhaft und kann höchstens eine leichte kurzzeitige Reizung der Haut im Gehörgang zur Folge haben.

Weiterhin würde bei Ihnen eine aktuelle Ton-Hörschwelle für die Frequenzen von 125 Hz bis 20 kHz ermittelt. Hierbei bekommen Sie über Kopfhörer Töne dargeboten, bei denen Sie angeben müssen, wann Sie diese wahrnehmen. Danach wird die sogenannte Unbehaglichkeitsschwelle bestimmt, bei der Sie angeben, ab welcher Lautstärke Ihnen ein Ton zu laut wird. Darauf folgt das Tinnitusmatching. Während der Messung bieten wir Ihnen verschiedene Töne und Geräusche in unterschiedlichen Frequenzen und Pegeln an, um herauszufin-

Universitätsklinikum Gießen und Marburg GmbH
Sitz der Gesellschaft: Gießen
Amtsgericht Gießen HRB 6384

www.ukgm.de

Geschäftsführung
Dr. Gunther K. Weiß (Vors.)
Prof. Dr. Werner Seeger (stv. Vors.)
Dr. Sylvia Heinis
Dr. Christiane Hinck-Kneip
Prof. Dr. Harald Renz

Aufsichtsratvorsitzender
Stephan Holzinger

den wie genau Ihr Tinnitus klingt.
Außerdem werden bei Ihnen otoakustische Emissionen (OAE) gemessen. Bei der Messung bekommen sie einen kleinen Einsteckhörer in den äußeren Gehörgang und hören dann Töne, bei denen Sie aber nicht angeben müssen, wann Sie diese wahrnehmen. Mit dieser Messung wird direkt die Funktion Ihres Innenohres überprüft. Zusätzlich würde noch eine sogenannte Tympanometrie durchgeführt, bei der man mit geringem Luftdruck die Beweglichkeit Ihres Trommelfells überprüft.
Die otologischen bzw. audiologischen Untersuchungsverfahren sind gänzlich nicht-invasiv. Unerwünschte Wirkungen dieser Untersuchungsverfahren sind nicht berichtet.

Die Dauer der audiologischen Messungen beträgt insgesamt ungefähr 45 Minuten.

Selbstverständlich ist die Teilnahme an dieser Studie freiwillig und Sie können jederzeit auch ohne Angabe von Gründen von der Teilnahme an der Studie zurücktreten. Hierdurch werden Ihnen keine Nachteile entstehen. Sämtliche Daten werden für die Studie pseudonymisiert und gespeichert. Das heißt, dass den von Ihnen erfassten Daten ein Personen-Code zugeordnet wird und alle Daten nur unter diesem Code gespeichert werden. Es existiert eine Zuordnungsliste, die Ihren Namen mit dem Code verbindet. Diese ist nur den Studienleitern zugänglich und wird nach Abschluss der Datenerhebung (spätestens zum 31.07.2021) vernichtet. Nach Vernichtung der Liste liegen Ihre Daten nur noch anonymisiert vor und sind nicht mehr Ihrer Person zuzuordnen. Die Daten werden ausgewertet, wissenschaftlich aufbereitet und auch publiziert.

Falls Sie noch weitere Fragen haben, können sie sich jederzeit melden bei:

Prof. Dr. Boris A. Stuck, Klinik für Hals,-Nasen- und Ohrenheilkunde, Universitätsklinikum Gießen und Marburg GmbH, Baldingerstraße, D-35043 Marburg

Dr. Katrin Reimann Klinik für Hals,-Nasen- und Ohrenheilkunde, Universitätsklinikum Gießen und Marburg GmbH, Baldingerstraße, D-35043 Marburg

Dr. Cornelia Weise (approbierte Psychotherapeutin), Philipps-Universität Marburg, Fachbereich Psychologie, AG Klinische Psychologie und Psychotherapie

Dr. Jochen Müller-Mazzotta Klinik für Hals,-Nasen- und Ohrenheilkunde, Universitätsklinikum Gießen und Marburg GmbH, Baldingerstraße, D-35043 Marburg

Kristina Sinemus Klinik für Hals,-Nasen- und Ohrenheilkunde, Universitätsklinikum Gießen und Marburg GmbH, Baldingerstraße, D-35043 Marburg

UNIVERSITÄTSKLINIKUM
GIESSEN UND MARBURG

Universitätsklinikum Gießen und Marburg GmbH • Klinik für HNO-Heilkunde
Baldingerstr., 35043 Marburg

STANDORT MARBURG
Klinik für Hals-, Nasen- und Ohrenheilkunde

Direktor:
Prof. Dr. Boris A. Stuck
Baldingerstr.
35043 Marburg
Telefon: 06421-5866478
Telefax: 06421-5866367
Mail:sekretariat.hno.mr@uk-gm.de
Internet: www.ukgm.de
Datum:
Zeichen: stuck-

Einwilligungserklärung der Probandin/des Probanden zur HNO-ärztlichen Untersuchung im Rahmen der Studie „Tinnitus und Neurofeedback“ (ToNe-Studie)

Ich habe die Probandeninformation gelesen und verstanden. Alle meine Fragen wurden ausreichend beantwortet und ich hatte ausreichend Zeit mich hinsichtlich der Teilnahme an der HNO-ärztlichen Untersuchung zu entscheiden.

Mir ist bewusst, dass die Teilnahme an der HNO-ärztlichen Untersuchung im Rahmen der ToNe-Studie freiwillig erfolgt und, dass ich die Studienteilnahme jederzeit ohne Angabe von Gründen und ohne Nachteile widerrufen kann. Die von mir für die Studie erhobenen Messdaten werden pseudonymisiert archiviert.

Mit der Unterschrift erteile ich meine Einwilligung zur Teilnahme an der HNO-ärztlichen Untersuchung im Rahmen der ToNe-Studie, zur Erhebung aller im Rahmen der Studie vorgesehenen Daten sowie zur Durchführung der geplanten Untersuchungen

bei mir: ……………………………………………….. (Nachname, Vorname)

Marburg, den ……………………………..

____
(Unterschrift des Probanden)

____
(Unterschrift aufklärender Arzt)

Universitätsklinikum Gießen und Marburg GmbH
Sitz der Gesellschaft: Gießen
Amtsgericht Gießen HRB 6384

www.ukgm.de

Geschäftsführung
Dr. Gunther K. Weiß (Vors.)
Prof. Dr. Werner Seeger (stv. Vors.)
Dr. Sylvia Heinis
Dr. Christiane Hinck-Kneip
Prof. Dr. Harald Renz

Aufsichtsratvorsitzender
Stephan Holzinger
