# Supplementary material for: Neurofeedback for tinnitus: study protocol for a randomised controlled trial assessing the specificity of an alpha/delta neurofeedback training protocol in alleviating both sound perception and psychological distress in a cohort of chronic tinnitus sufferers
Source: Trials. 2020 May 5;21:382. doi: 10.1186/s13063-020-04309-y (PMC7201543; doi:10.1186/s13063-020-04309-y)
Supplement: Supplementary file 6 — Additional file 6. Participant Information regarding the ENT assessment part of the study "Tinnitus and Neurofeedback" (ToNe study). [file 13063_2020_4309_MOESM6_ESM.docx]

**Participant Information regarding the ENT assessment part of the study "Tinnitus and Neurofeedback" (ToNe study).**

Dear participant,

We would like to inform you about the above study:

Regarding background, questions and methodical approaches within the study, we refer to the information sheets of the Department of Psychology, AG Clinical Psychology and Psychotherapy, Philipps-University Marburg, which you have already read.

The study requires that you have an otoscopy and, if necessary, a cleaning of the ear canal. In this case an ENT-medical check-up is performed and if necessary, any cerumen (“ear wax”) gently removed. This is neither painful, nor invasive, and at most, may cause a slight short-term irritation of the skin in the ear canal. Furthermore, we will determine your hearing threshold for the frequencies from 125 Hz to 20 KHz. Sounds will be presented in headphones and you must indicate, when you perceive them. Following this, the so-called discomfort threshold is determined. Here, you will specify, at what level, the sound is too loud for you. This is followed by tinnitus matching. During this assessment, we present you with different tones and noises at different frequencies to identify, exactly what your tinnitus sounds like. In addition, we measure otoacoustic emissions (OAE). In this measurement, a small plug is inserted in the outer ear canal and sound is played. You do not have to specify, when you perceive them. This is a measurement to test the function of your inner ear. With the so-called Tympanometry, we check the working of your ear drum with low air pressure.

The otological and audiological examination procedures are completely non-invasive. No undesirable effects of these examinations have been reported. The duration of the audiological assessment is approximately 45 minutes. Participation in this study is voluntary and you can always withdraw from participation in the study without giving any reason. That is, you will not experience any disadvantages as a result of withdrawal from the study.

All data collected are pseudonymized. This means that the data collected from you is assigned a person code and data stored under this code. There is an assignment list, which connects your name with the code. This is only accessible to the members of the study group and will be deleted after completion of the data collection (by 31.07.2021 at the latest). Upon deletion of the list, your data are only available anonymously and can no longer be matched to your person. The data is subject to scientific evaluation and publication.

If you have any further questions, you can always contact:

- Prof. Dr. Boris A. Stuck, Department of Otolaryngology, University Hospital

Gießen and Marburg GmbH, Baldingerstraße, D-35043 Marburg

- Dr. Katrin Reimann Department of Otolaryngology, University Hospital

Gießen and Marburg GmbH, Baldingerstraße, D-35043 Marburg

- Dr. Cornelia Weise (certified psychotherapist), Philipps-University Marburg,

Department of Psychology, WG Clinical Psychology and Psychotherapy

- Dr. Jochen Müller-Mazzotta Department of Otolaryngology, University Hospital

Gießen and Marburg GmbH, Baldingerstraße, D-35043 Marburg

- Kristina Sinemus Department of Otolaryngology, University Hospital

Gießen and Marburg GmbH, Baldingerstraße, D-35043 Marburg

**Informed consent.**

I am aware that participation in the ENT examination, as part of the ToNe Study is voluntary and that I can withdraw from the study at any time without giving a reason. The data collected from me during the assessment are pseudonymized.

With my signature, I consent to participate in the ENT medical examination under the framework of the ToNe study. This consent includes the collection of data and the execution of the planned examinations.

My signature: …………………. (Surname, Name)

Marburg, date …………………

Signature participant

Signature Audiological assessor
