# Supplementary material for: Neurofeedback for tinnitus: study protocol for a randomised controlled trial assessing the specificity of an alpha/delta neurofeedback training protocol in alleviating both sound perception and psychological distress in a cohort of chronic tinnitus sufferers
Source: Trials. 2020 May 5;21:382. doi: 10.1186/s13063-020-04309-y (PMC7201543; doi:10.1186/s13063-020-04309-y)
Supplement: Supplementary file 7 — Additional file 7. Trial registration—dataset. [file 13063_2020_4309_MOESM7_ESM.docx]

| **Trial registration – data set** | |
| --- | --- |
| **Data category** | **Information** |
| Primary registry and trial identifying number | NCT03550430 |
| Date of registration in primary registry | 08. Juni 2018 |
| Secondary identifying numbers | N/A |
| Source(s) of monetary or material support | The Oticon Foundation has funded one PhD position in addition to costs of procuring equipment, recruiting and re-imbursing participants. The funding source has no role in the design of this study and will have no role during its execution, analyses, interpretation of the data or in the decision to submit results. |
| Primary sponsor | Philipps Marburg  Klinische Psychologie & Psychotherapie  Fachbereich Psychologie  Address: Gutenbergstraβe 18, DE-35032 Marburg  Contact: Dr. Cornelia Weise  Email: cornelia.weise@staff.uni-marburg.de |
| Secondary sponsor(s) | Eriksholm Research Centre  Rørtangvej 20, DK-3770 Snekkersten  Contact: Senior Director Uwe Andreas Hermann  Email: uwhe@eriksholm.com |
| Contact for public queries | Contact: Dr. Cornelia Weise  Email: cornelia.weise@staff.uni-marburg.de |
| Contact for scientific queries | Contact: Dr. Cornelia Weise  Email: cornelia.weise@staff.uni-marburg.de |
| Public title | Study protocol for a randomised controlled trial assessing the specificity of an alpha/delta neurofeedback training protocol in alleviating both sound perception and psychological distress in a cohort of chronic tinnitus sufferers. |
| Scientific title | Study protocol for a randomised controlled trial assessing the specificity of an alpha/delta neurofeedback training protocol in alleviating both sound perception and psychological distress in a cohort of chronic tinnitus sufferers. |
| Countries of recruitment | Germany |
| Health condition(s) or problem(s) studied | Chronic tinnitus |
| Intervention(s) | Alpha/delta neurofeedback training protocol (active comparator)  Beta/theta neurofeedback training protocol (control group) |
| Key inclusion and exclusion criteria | Inclusion criteria: adults > 18, chronic, subjective tinnitus with a duration of ≥ 6 months, tinnitus distress ≥ 18 on THI  Exclusion criteria: objective, tinnitus, severe mental health issues, use of psychotropic drugs, substance abuse, neurological conditions, prior experience with neurofeedback, current treatment for tinnitus |
| Study type | Interventional, double-blind study with randomized allocation.  Primary purpose: treatment |
| Date of first enrolment | 1. November 2018 |
| Target sample size | 98 |
| Recruitment status | Recruiting |
| Primary outcome(s) | Reduced distress associated with tinnitus  Reduced intensity of tinnitus percept |
| Key secondary outcomes | Changes in EEG frequency bands |
